# Adding Chemical Cross-Links to a Physical Hydrogel

**DOI:** 10.3390/molecules14093662

**Published:** 2009-09-17

**Authors:** Gaio Paradossi, Ivana Finelli, Barbara Cerroni, Ester Chiessi

**Affiliations:** Dipartimento di Scienze e Tecnologie Chimiche, Università di Roma Tor Vergata and CNR – SOFT, Italy; E-Mails: ivana.finelli@uniroma2.it (I.F.); b.cerroni@gmail.com (B.C.); ester.chiessi@uniroma2.it (E.C.)

**Keywords:** hydrogels, swelling, biocompatibility, polysaccharides

## Abstract

Synergistic hydrogels are often encountered in polysaccharide mixtures widely used in food and biopharma products. The xanthan and konjac glucomannan pair provides one of the most studied synergistic hydrogels. Recently we showed that the junction zones stabilizing the 3D structure of this gel are present as macromolecular complexes in solution formed by the partially depolymerised polysaccharidic chains. The non-covalent interactions stabilizing the structure of the polysaccharidic complex cause the melting of the ordered structure of the complex in the solution and of the hydrogels. Introduction of chemical cross-links in the 3D structure of the synergistic hydrogel removes this behaviour, adding new features to the swelling and to the viscoelastic properties of the cured hydrogel. The use of epichlorohydrin as low molecular weight cross-linker does not impact unfavourably on the viability of NIH 3T3 fibroblasts.

## 1. Introduction

Xanthan, a microbial polysaccharide produced by the bacterium *Xanthomonas campestris*, has the remarkable feature of forming physical hydrogels when it is mixed with gluco- or galactomannans [1,2,3]. These hydrogels are usually called “synergistic gels” as their formation involves specific interactions between these two types of polysaccharides, whereas the mixing of xanthan with other polysaccharides at similar concentrations does not provide a gel phase.

This effect indicates that a specific interaction pattern occurs between the chains of xanthan with either glucomannan, a copolymer of 1,4-linked β-D-mannose and β-D-glucose, or galactomannan, a 1,4-linked β-D-mannose with α-D-galactose side-chains [2,4]. Investigations on hydrogels based on the combined presence of xanthan, or differently modified xanthan, and several other (bio)polymers demonstrate the interest in adding new features to materials with known performances. Poly(acrylamide-grafted-xanthan)-based pH-sensitive hydrogel beads have been considered for enteric delivery of ketoprofen [5]

In a previous paper [6], we have studied the solution behaviour of the depolymerised xanthan and konjac glucomannan; hereafter named KGM, a complex where the involvement of side-chains appeared as a relevant factor for the establishment of the macromolecular interactions. Similar conclusions were drawn in an earlier former paper by Annable *et al*. [7] on the basis of DSC and EPR experiments indicating in the reduction of the xanthan-water contacts the driving force stabilizing the synergistic hydrogel. 

In this work we describe the effect of introducing chemical cross-links in the synergistic hydrogels in order to add new properties to the starting xanthan-KGM hydrogels. As expected based on rubber elasticity theory [8], the swelling behaviour, the storage and loss moduli will be affected by such modification, as well as the hampering of the hydrogel melting. Addition of chemical cross-links to the xanthan–KGM physical network was accomplished using epichlorohydrin, a very reactive difunctional molecule often recognized for its toxicity. However, it should be considered that epichlorohydrin is presently used as additive in paper products for food industry and in water treatment [9]. We report here on two xanthan/konjac glucomannan hydrogels, hereafter named XGE1 and XGE2 cured with different epichlorohydrin concentrations, *i.e.,* 0.6 M and 1.4 M, and the impact of epichlorohydrin used as cross-linker on the viability of fibroblasts NIH 3T3 seeded on the chemically cross-linked hydrogels was assessed.

## 2. Results and Discussion

**Figure 1:**
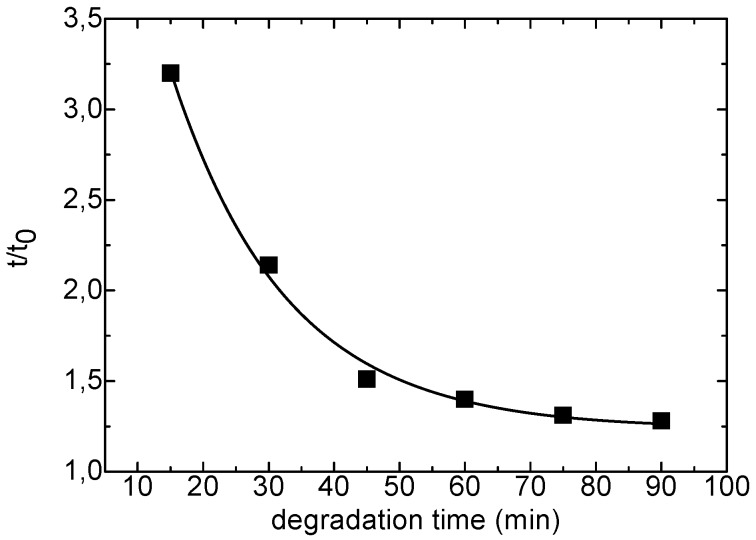
Enzymatic degradation of KGM by cellulase.

The characterization of xanthan and KGM polysaccharides was performed on intact and partially depolymerised samples. For the study in solution glucomannan was hydrolyzed with cellulase having endo-activity for the β(1→4)glucosic linkages. The chain degradation was carried out in acetate buffer at pH 4.5 at 37 °C in the presence of 0.1% (w/v) enzyme and monitored by measuring the shear times of the KGM solution in a Ubbelhode suspended flow capillary viscosimeter. After 1 hour the degradation was completed, with a 3-fold drop in the relative viscosity, t/t_0_, as shown in Figure 1.

**Figure 2:**
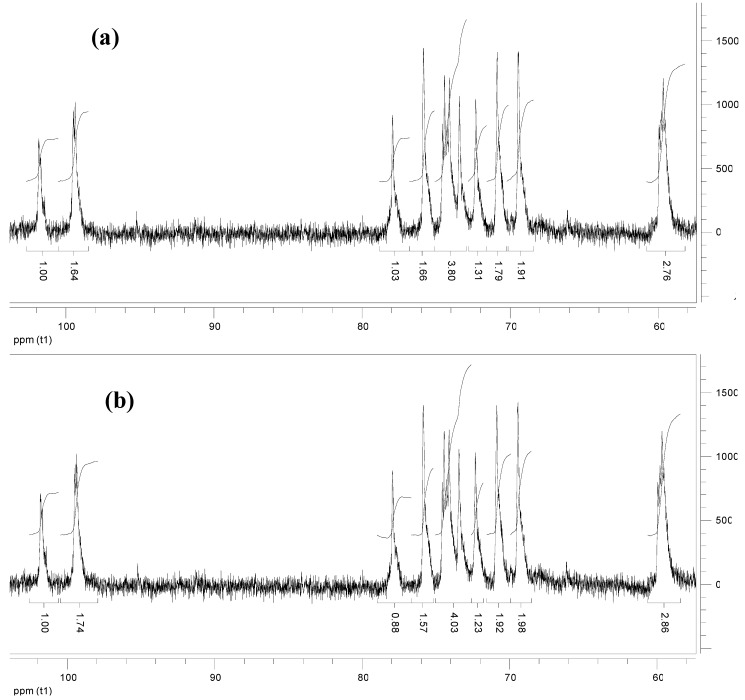
^13^C-NMR spectra of (a) KGM after 10 min degradation and (b) KGM after 60 min degradation.

Whether the enzyme degradation affects the chemical structure of depolymerised KGM was addressed by ^13^C-NMR. Figure 2 shows the ^13^C-NMR spectra of KGM samples degraded for 10 (spectrum a) and for 60 minutes (spectrum b). Assignment of the resonances was based on the literature [6,10]. Integration of peaks at 101.7 and at 104 ppm, relative to non-reducing anomeric carbons of mannose and glucose, respectively, gives a value of the mannose:glucose ratio of 1.7. As ^13^C-NMR is not commonly used for quantitative determinations, this result was validated by ^1^H-NMR (not shown) giving similar results. The same mannose:glucose ratio was also found for native KGM.

**Figure 3:**
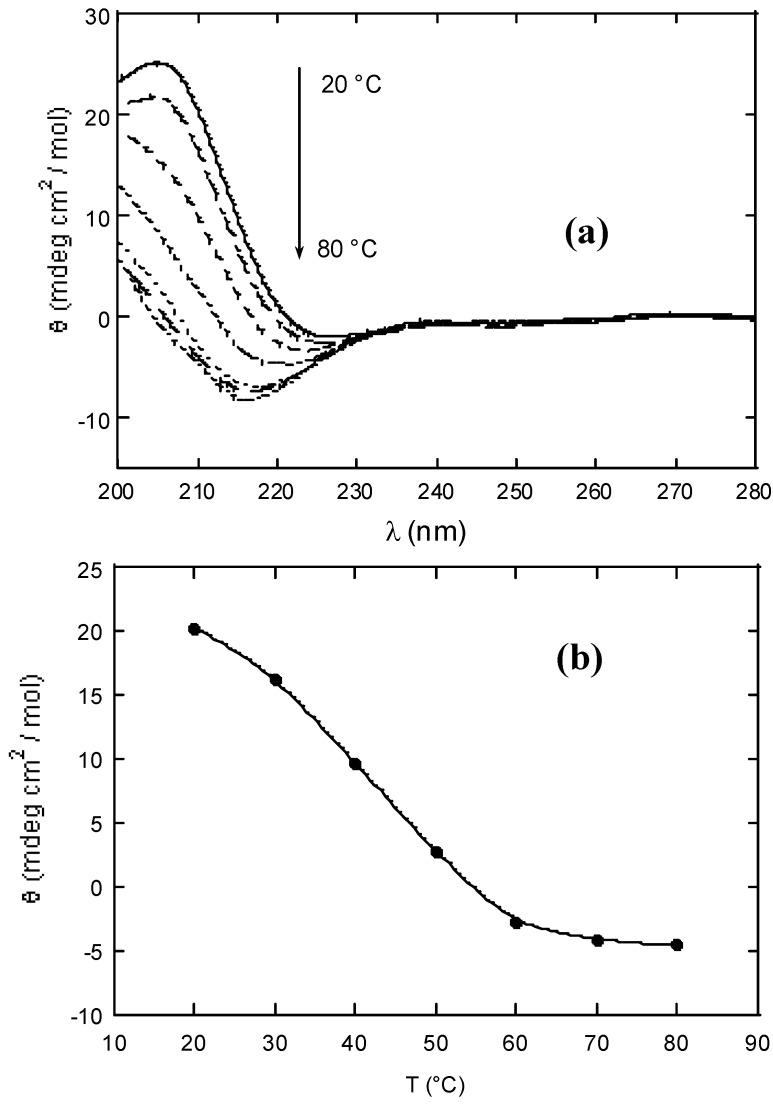
(a) Circular dichroism spectra of partially depolymerised xanthan-KGM (1:1 w/w) aqueous solution (b) and ellipticity at 210 nm (b) as a function of temperature (step 10 °C).

Xanthan was depolymerised by high power sonication for four hours. Molecular parameters for depolymerised xanthan and KGM polysaccharides are summarized in Table 1. The interaction between xanthan and KGM in water was monitored by circular dichroism, CD, on partially depolymerised chains, as the use of native (intact) polymers at the investigated concentrations is not suitable for CD study due to sudden formation of the gel phase. The contribution of KGM to the total ellipticity is negligible. As the carboxylic groups beared in the xanthan side-chains are the CD chromophores, the spectrum reflects the conformational state of xanthan side-chains [6] complexing the glucomannan. A melting curve with midpoint at about 45 °C shows an order ↔ disorder transition revealing that the synergistic interaction is coupled with the ordering of xanthan side-chains (see Figure 3).

**Table 1:** Molecular parameters of Xanthan and KGM.

Xanthan sonicated 4h	Native KGM	KGM degraded 10min
M_w_ = 620,000 g/mol	M_w_ = 960,000 g/mol	M_w_ = 560,000 g/mol
A_2_ = 6.7·10^-04^ cm^3^mol/g^2^	A_2_ = 3·10^-05^ cm^3^mol/g^2^	A_2_ = 4.3·10^-05^ cm^3^mol/g^2^
Rg = 98 nm	Rg = 100 nm	Rg = 70 nm
dn/dc = 0.17 mL/g	dn/dc = 0.15 mL/g	dn/dc = 0.15 mL/g

**Figure 4:**
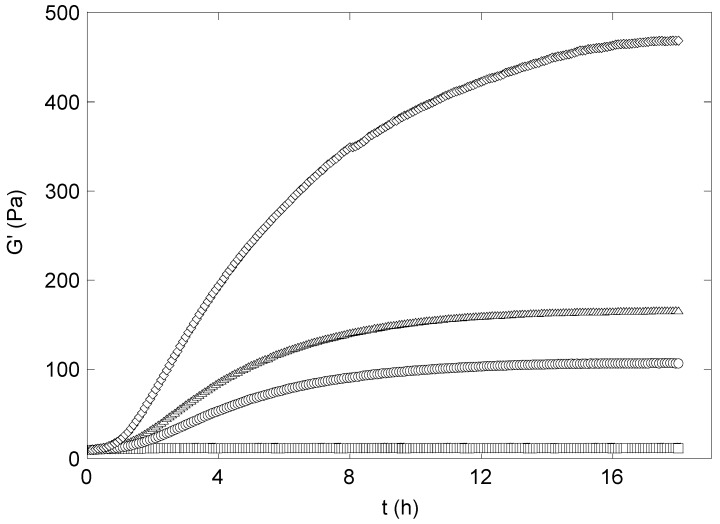
Kinetics of the cross-linking of xanthan-KGM mixture by epichlorohydrin. Initial cross-linker concentration: (□) 0.1 M; (○) 0.3 M; (△) 0.6 M; (◊) 1.4 M.

The gel formation was monitored by small strain oscillatory shear measurements, recording the time dependence of G’ and G”. The time dependence of G’ in synergistic xanthan/KGM hydrogels during the curing with epichlorohydrin was monitored at 1 Hz (see Figure 4). Storage modulus was always higher than the loss modulus, a feature indicating clearly that the cross-linking reaction starts and is carried out in an existing synergistic gel phase provided by the mixing of the two polysaccharides. G’ shows the typical trend of a cross-linking reaction: An induction time, present for any amount of epichlorohydrin, although the extent of this phase depends on the cross-linker concentration, followed by a stage where G’ increases linearly, and a constant asymptotic behaviour at long times. 

The swelling and elastic properties of the synergistic hydrogels stabilized by chemical cross-links depend on the amount of polymer effectively incorporated in the hydrogel contributing to the osmotic (included Donnan) and elastic terms of water activity. The gel and the soluble amounts, W_gel_ and W_sol_ (see Experimental section for definitions), account for the effective incorporation of the chains of the two polysaccharides in the network. 

**Figure 5:**
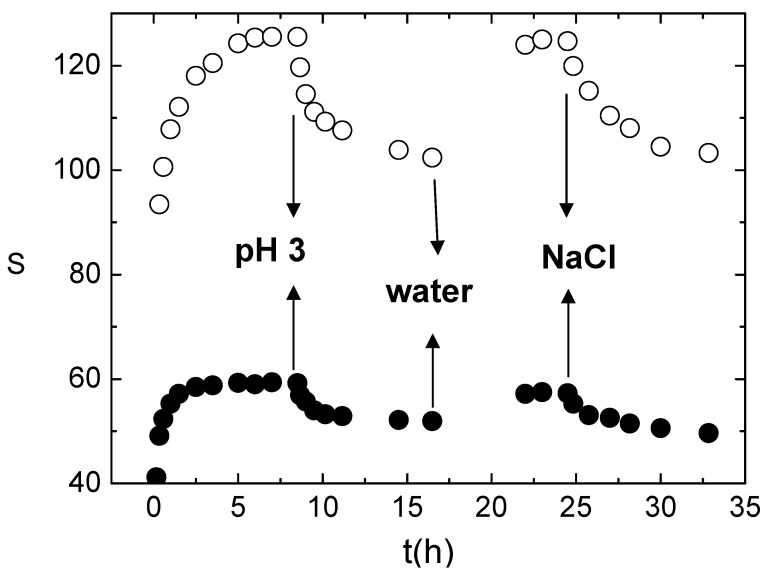
Swelling/de-swelling behaviour: water weight fraction, S, in XGE hydrogels stored sequencially in HCl 10^-3^ M, water, 0.1M NaCl. Empty symbols: XGE1; full symbols XGE2.

Introduction of chemical cross-links adds new features to the hydrogels. The water weight fraction, S, defined as (W_s_ – W_gel_)/W_gel_ with W_s_ representing the weight of swollen hydrogel (see Experimental section) was monitored at different times during the swelling/de-swelling process of the hydrogel in a pH cycle, revealing the polyelectrolyte behaviour of xanthan chains in the hydrogel, as shown in Figure 5. A responsive behaviour to ionic strength changes, as a consequence of the Debye screening effect of the xanthan charges confined in the hydrogel, is also detected when the hydrogel is equilibrated in 0.1 M NaCl.

Storage modulus, G’, of chemically cross-linked synergistic hydrogels was used to assess the density of cross-links contributing to the elasticity of the system. According to modified rubber elasticity theory [8,11] to take into account that the cross-linking reaction was carried-out in the “nascent” state before the swelling process, the cross-links density in this state, ν_e_/V_0_, is related to G’ according to equation 1:

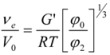
(1)
where φ_0_ and φ_2_ are the polymer volume fractions in the relaxed state, i.e. after cross-linking and before the swelling, and after the swelling, respectively, and Q is the degree of swelling defined as (1/φ_2_). Equation 1 holds for affine networks, i.e. ideal networks where all cross-links have the same contribution to the macroscopic elasticity and chain deformations are the same as the macroscopically imposed strain. In Table 2 the swelling features of the hydrogels are summarized in water, at pH 3 and in NaCl 0.1 M.

**Table 2:** Swelling parameters of XGE hydrogels.

Hydrogel	W_gel_(g)	W_0_(g)	W_sol_(g)	W_s_(g)	φ_0_	φ_2_	Q
XGE1	0.042	0.16	0.12	5.4	0.015	0.006	167
XGE2	0.055	0.31	0.26	3.3	0.020	0.013	77
XGE1 pH 3	0.042	0.16	0.12	4.4	0.015	0.007	143
XGE2 pH 3	0.055	0.31	0.26	3.0	0.020	0.014	71
XGE1 NaCl 0.1M	0.068	0.16	0.092	4.4	0.025	0.011	91
XGE2 NaCl 0.1M	0.073	0.31	0.24	3.1	0.026	0.018	56

The rheology behaviour of the hydrogels, summarized in Table 3, provides information about the elastic active chains in the hydrogel.

**Table 3:** Storage modulus and chain density in XGE hydrogels.

Hydrogel	G’(Pa)^a^	ν_e_/V_0_	ν_e_/V	(ν_e_/V)_th_^b^
		·10^6^(mol·cm^-3^)	·10^6^(mol·cm^-3^)	·10^3^(mol·cm^-3^)
XGE1	1180	0.669	0.271	0.448
XGE2	2270	1.086	0.762	1.728
XGE1 pH 3	780	0.191	0.102	0.550
XGE2 pH 3	1500	0.696	0.526	1.898
XGE1 NaCl 0.1M	1050	0.546	0.250	0.551
XGE2 NaCl 0.1M	395	0.186	0.136	1.84

^a^ determined at 1 Hz; ^b ^values based on the amount of cross-linker added initially.

Inspection (see Table 3) of the values of chain density in the relaxed state (before swelling), ν_e_/V_0_, obtained from the storage modulus according to the affine model of the rubber elasticity theory (Equation 1), of the chain density values of the swollen hydrogel, of the chain density in the swollen state, ν_e_/V, and of the theoretical (ν_e_/V)_th_ based on the epichlorohydrin initially added (see Experimental) shows that the elastically effective crosslinks in the network are about three orders of magnitude lower than the amount of epichlorohydrin initially added to the hydrogel. 

The mass of epichlorohydrin converted into cross-links is about 0.4% and 0.5% of the total dry weight in XGE1 and XGE2, respectively. The molecular weight between cross-links can be estimated according to equation (2):

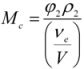
(2)
as about 32,000 and 23,000 g/mol, corresponding to 180 and 130 sugar residues, for XGE1 and XGE2, respectively. 

**Figure 6:**
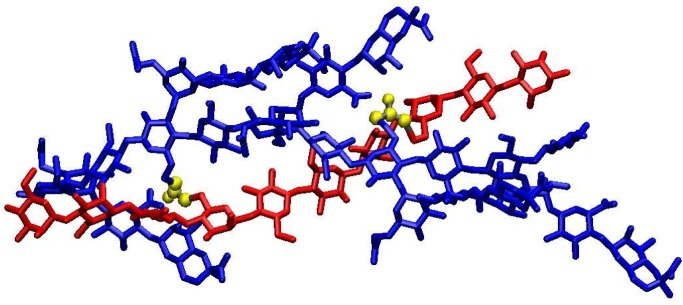
Molecular representation of the junction domain in XGE hydrogel. Epichlorohydrin cross-links are evidenced in yellow. Xanthan and glucomannan chains are shown in blue and red coloros, respectively. Hydrogen atoms are not shown.

A molecular representation of the junction domain in the hydrogel can be obtained by considering that cross-linking by epichlorohydrin occurs on the xanthan-KGM network of the synergistic hydrogel. Therefore it can be supposed that the cross-linker molecules will join the two polysaccharide chains in sterically favourable positions inside the regions connected by physical junction, already present before the curing. The stoichiometry of the complex is about 1:1 in mass and two compatible models of the macromolecular assembly can be inferred, considering either a linear or a branched KGM. By considering the un-substituted KGM, a 1:2 macromolecular complex (one xanthan chain: two KGM chains) is obtained, whereas, by assuming 10% as maximum side-chain substitution degree, a 1:1 assembly (one xanthan chain: one KGM chains) is obtained. For both complexes we built, by molecular mechanics, models stabilized by intermolecular hydrogen bonds and Van der Waals interactions [6]. During the synthesis of XGE hydrogels, chemical bridges are placed inside the macromolecular complexes, involving the more reactive saccharide hydroxylic groups. The junction domain structure for the 1:1 xanthan:KGM assembly is shown in Figure 6, where a decasaccharide of KGM and a xanthan chain of five repeating units are bound by two epichlorohydrin bridges (yellow atoms) between C6 carbon atoms of the KGM and of xanthan backbone chains. The complex organizes in a double helical arrangement with antiparallel chains orientation and the average distance between backbones is about 8 Å. The cross-linking degree shown in Figure 6 (one epichlorohydrin every 18 sugar residues) does not reproduce the experimental picture as the image provides a description of the junction with an atomic detail in a spatial domain of few nanometers. The presence of chemical cross-links in XGE gels increases the hydrogel responsivity to sharp gradients of external parameters as pH and ionic strength and allows a reversible behaviour of the system.

**Figure 7:**
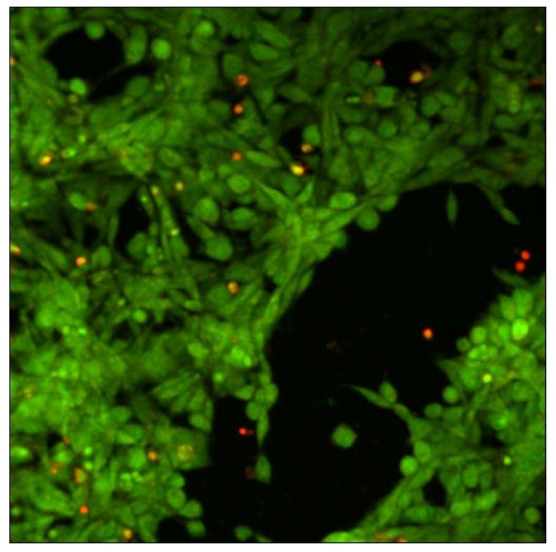
Laser scanning confocal microscopy image of “live - dead” viability test on NIH 3T3 fibroblasts.

Cytotoxicity of xanthan/KGM chemically cross-linked synergistic hydrogels was evaluated by seeding murine fibroblasts NIH 3T3 on the surface of XGE1, cured with 0.6 M epichlorohydrin. Cell viability was tested with “Live-Dead” assay. In this test live cells display a green colour in fluorescence microscopy mode, whereas red stained cells indicate dead cells. Figure 7 illustrates the fibroblast viability after 12 h incubation on the surface of XGE1 hydrogels in the presence of a clear predominance of live cells is detected with the typical morphology of adhered NIH 3T3 cells. It can be concluded that the low epichlorohydrin content acting as cross-linker in the hydrogel, is not unfavourable to cells viability as a very low cross-linking density is enough to add responsivity features to physical synergic hydrogels.

## Conclusions

Introduction of chemical cross-links in xanthan – konjac glucomannan synergistic hydrogel adds new features as the response to external gradients of pH and ionic strength and the enhanced mechanical behaviour. These properties can be an asset for application of these materials in biomedicine. Chemical junctions were introduced by curing the hydrogels with epichlorohydrin, a bifunctional cross-linker often used for polysaccharides coupling. The impact of epichlorohydrin cured polysaccharide hydrogels on cell viability was assessed by incubating fibroblasts with chemically cured xanthan glucomannan synergistic hydrogels, showing that these systems can be used in the presence of cell materials with additional potentialities offered by the presence of chemical cross-links.

## Experimental

### Materials

Glucomannan from *Amorphophallus konjac*, hereafter called KGM, and xanthan gum (trade name Keltrol) from *Pseudomonas campestris* were supplied by Marine Colloids Division of FMC Corp. and Kelco International Ltd., respectively. Determination of acetyl and pyruvate content in xanthan, carried out by proton NMR, gave a degree of substitution of 0.9 and 0.6, respectively. Degradation of KGM was carried out by using cellulase (EC 3.2.1.4) from *Trichoderma viride* (Sigma). 

Inorganic salts, buffer solutions, sodium EDTA were Carlo Erba products and used without further purification. Deionized water with conductivity less than 18.2 MΩ cm was produced with a USF Elga water purifier. Dialysis membranes with a cut off of 12,000 kDa were supplied by Sigma. A micro glass electrode (Amel) was used for pH determinations.

*Xanthan solutions*: Native xanthan was degraded by sonication using a VCX 400 sonicator (Sonics and Materials Inc.) with a maximum output power of 400 W at a frequency of 20 kHz equipped with a macrotip (TI-6AL-4V). The sonication was carried out with a sequence of 1 s pulse and 0.3 s standby at approximately 80% of the maximum power. Five hundred mL batches of 0.6% (w/v) solution of xanthan were added with 1% (v/v) of acetone as radical scavenger and solid NaCl to a concentration of 0.1 M. Sonications were carried out in an external water/ice bath in order to avoid overheating. After sonication, solutions were centrifuged at 10,000 rpm at 2 °C for 1 h with a Beckman J2-21 ultracentrifuge equipped with a J14 rotor in order to separate titanium particles from tip erosion and cell debris. Solutions were dialyzed repeatedly for one day against 0.03 M EDTA and then exhaustively for a week against Milli-Q grade water at 4 °C. Xanthan solutions were stored at 4 °C at a concentration of 5% (w/v). Before mixing with glucomannan solutions, xanthan was always heated at 60 °C for 1 hour.

*Glucomannan solutions*: Konjac glucomannan, hereafter indicated as KGM, was dispersed at a concentration of 0.5 (w/v) in 0.1 M sodium acetate/acetic acid buffer at pH 4.5 under vigorous mechanical stirring and autoclaved for 20 min at 120 °C. 

### Enzymatic degradation of KGM

To characterize the sugar composition of KGM, an enzymatic degradation was carried out as the viscosity of the sonicated samples made impracticable the analysis by ^13^C-NMR spectroscopy. The hydrolytic activity of cellulase on the β(1→4) glycosidic linkages of nearest neighbor glucose units allowed a degradation of KGM polymer chains to oligosaccharides. Some β(1→4) mannanase activity was also observed in this enzyme preparation. A typical enzymatic degradation was carried out as follows: 0.4% (w/v) KGM in sodium acetate/acetic acid buffer 0.1 M at pH 4.5 was added at 37 °C with an amount of enzyme equal to 0.1 of the polysaccharide mass. Enzyme was incubated at 37 °C for 1 h before addition to the polysaccharide solution. Enzymatic digestion was carried out for 10 min and for 1 h and stopped by adding 2-propanol to a concentration of 60% (v/v) in order to precipitate the saccharide moiety. The precipitated xanthan was washed with 2-propanol and dried. 

^13^C-NMR determination was carried out with a Brüker AM400 working at 100.63 MHz on 5% (w/v) solutions of degraded KGM in deuterated DMSO. Chemical shifts were determined with respect to dimethylsulfoxide. The information directly obtained from the C1 region of the degraded KGM spectra is based on the assignments available in the literature for glucomannan from cell walls of endosperm of *A. officinalis* [7]. The resonances grouped around 104 ppm and around 101.6 ppm were assigned to the nonreducing anomeric carbons of α-D-glucose residues and of the α-D-mannose residues, respectively. C1 resonances of reducing glucose and mannose units ranged from 93.4 to 97.4 ppm. On this basis it was possible to evaluate the ratio mannose/glucose of KGM. 

### Circular dichroism

The spectra were recorded with a JASCO J600 spectropolarimeter in the UV range 200-280 nm with quartz cells with an optical path of 0.5 and 0.1 cm. Temperature was controlled with a LAUDA M3 thermostat.

### Light scattering

A BIC200 photometer (Brookhaven, USA) equipped with a solid state laser emitting at 532 nm was used in an angular range from 20 to 154 degrees. Decalin was used as refractive index matching liquid. Measurements were carried out in 0.1 M NaCl with polymer concentrations ranging from 0.1 mg/mL to 7 mg/mL for sonicated xanthan, from 0.2 mg/mL to 3.5 mg/mL for native KGM and from 1 mg/ml to 10 mg/mL for degraded KGM. Toluene was used as reference liquid using a Rayleigh ratio, R_T_ = 2.6 × 10^-5^ cm^-1^ at 532 nm. The polymer solution Rayleigh ratio, R_θ_, was determined as:

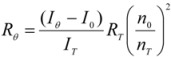
(3)
where I_θ_ is the scattering intensity of the sample at angle θ, I_0 _is the scattering intensity of the solvent, I_T _is the scattering intensity of toluene with the refractive indexes of the solvent and of toluene, n_0_ and n_T__,_ respectively.

The optical constant 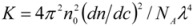, where λ is the laser radiation wavelength, was determined by measuring the differential refractive index, (dn/dc), by means of a BIC DNDC (Brookhaven, USA) calibrated with KCl aqueous solutions at 532 nm. The molecular parameters reported in Table 1 were extracted according to the data treatment suggested by Zimm [12]:

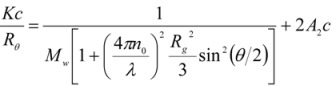
(4)
where c is the polymer concentration (w/v) and M_w_, R_g_ and A_2 _are the weight average molecular weight, the radius of gyration and second virial coefficient of the polymer, respectively.

### Rheology measurements

Capillary viscosimetry measurements were carried out at 25 °C by using a suspended-flow viscosimeter (Ubbelhode type) with a flow time for water of 40 s. Dynamic viscoelastic measurements were performed with a AR 2000 Advanced Rheometer (TA Instruments) using a parallel plates geometry (diameter 20 mm, gap 2 mm) with the fixed plate equilibrated at 25 °C. Hydrogels were poured directly onto the plate of the instrument.

1. Two types of viscoelastic measurements were carried out:
  deformation sweeps at a constant frequency (1Hz) to determine the maximum deformation attainable by a sample in the linear viscoelastic range.
2. storage and loss moduli, G’ and G”, on swollen hydrogels were obtained in small strain oscillatory shear. An amplitude sweep (G’ vs. strain) was performed in order to assure that the measurements were carried out in the linear viscoelastic regime. The mechanical spectra were obtained recording the dynamic moduli G’and G” in requency sweeps at a constant deformation, 1% strain (limit of linear viscoelastic strain was about 10%).

### Formation and characterization of chemical hydrogels

Hydrogels were synthesized with native xanthan gum and KGM by mixing a 1:1 (w/w) ratio of polysaccharides to a total polymer concentration of 2 wt% in an aqueous solution containing 1.5 M NaOH and 3.3 M urea. The mixture was stirred at 25 °C for 1h and then was stored at -4 °C for 18h. The frozen solid was thawed and stirred at room temperature for 30 min. A given amount of epichlorohydrin (0.6 M and 1.4 M), used as crosslinker, was added dropwise into the solution. The mixture was stirred for 10 min and stored at room temperature for 72 h. The reaction mixture was then neutralized with acetic acid, washing exhaustively with 1:3 (v/v) water/acetone and finally with water. 

The amount of polymer participating to the network formation, W_gel_, was determined by drying the hydrogel after exhaustive washings. Correspondingly, W_sol_, was evaluated according to W_sol_ = W_gel_ – W_0, _where W_0_ is the initial mass of polysaccharides used in the chemical cross-linking. 

Swelling degree was evaluated according to Q= W_s_/W_gel_ where W_s_ is the weight of the swollen gel. 

The theoretical values of the chain density,(ν_e_/V)_th_, based on the epichlorohydrin initially added, were worked out according to the equation:

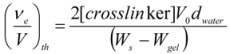

where d_water_ is the density of water and V_0_ is the volume of the gel before the cross-linking reaction, typically 2 mL.

Swelling/deswelling cycles were carried out by equilibrating the hydrogels, typically 5 g, in a large volume reservoir containing the aqueous medium with required characteristics of pH or ionic strength under gentle stirring, in the sequence: water, pH 3, water, 0.1 M NaCl.. Weight of the swollen gel, W_s_, was determined at different times during the cycles.

### Molecular modelling

A molecular model of the cross-linked domain was obtained by the molecular viewer software package VMD [13], starting from an energy minimized structure of the xanthan-KGM 1:1 complex [6] and by adding epichlorohydrin bridges in sterically allowed positions.

### Cell cultures

NIH 3T3 mouse fibroblasts from the Istituto Sperimentale Zooprofilattico della Lombardia e dell’Emilia Romagna (Italy), were cultured in Dulbecco’s Modified Eagle Medium (DMEM) high glucose, supplemented with 10% fetal bovine serum, 20 mM of L-Glutamine, 100 U/mL penicillin and 100 µg/mL streptomycin at 37 °C in a 5% CO_2_. 

Hydrogels XGE1 and XGE2 were inserted in multi-well plate (12 wells) and covered with DMEM to permit the exchange between water and cell medium. DMEM was replaced twice and then gels were sterilized by 30 min UV exposure. 

Approximately 700,000 NIH 3T3 cells were seeded on gels and allowed to settle 12 hours prior observation by light microscopy (Nikon Eclipse TE 2000-S) to check if cells were able to adhere on the surface of XGE1 and XGE2 hydrogels.

### Cell viability studies

*Live or dead assay*: Live/Dead^®^ Reduced Biohazard Viability/Cytotoxicity Kit (Molecular Probes) was used to assess cell viability and cytotoxicity. This assay permitted us to distinguish metabolically active cells from injured and dead cells. After washing cells with HBSS solution (135 mM NaCl, 5 mM KCl, 1 mM MgSO_4_, 1.8 mM CaCl_2_, 10 mM HEPES, pH 7.4), they were incubated for 15 min at room temperature protected from light with a solution containing 1:500 dilution of each probe provided in the kit, SYTO 10 green fluorescent nucleic acid stain and DEAD Red (ethidium homodimer-2) nucleic acid stain. After incubation, cells were washed with HBSS and fixed with 4% glutaraldehyde in HBSS for 1h at room temperature, in the dark. Excess of glutaraldehyde was removed and it was replaced by HBSS. Green and red stained cells were observed under a confocal laser scanning microscope, Nikon Eclipse TE 2000-S equipped with a He-Ne and a Ar^+^ lasers. 
